# HAM/TSP patient treated with porcine cortex monosialoganglioside: clinical outcome and follow up

**DOI:** 10.1186/1742-4690-12-S1-P27

**Published:** 2015-08-28

**Authors:** Camila Cánepa, Jimena Salido, Gabriela Pataccini, Matías Ruggieri, Sindy Fraile, Carolina Berini, Mirna M Biglione

**Affiliations:** 1 Instituto de Investigaciones Biomédicas en Retrovirus y SIDA (INBIRS), UBA-CONICET, Buenos Aires, Argentina

## 

HTLV-1 infects about 20 million people worldwide and is considered the etiologic agent of Adult T-cell Leukemia/Lymphoma (ATLL) and HTLV-1 Associated Mielopathy/Tropical Spastic Paraparesis (HAM/TSP). There is still no effective therapy for none of the pathologies. Porcine cerebral cortex GM1 monosialoganglioside (SINAXIAL®) is being prescribed for traumatic or acute peripheral neuropathies, acute cerebrovascular and others. CD4 modulation and inhibition of HIV-1 infectivity induced by monosialoganglioside GM1 in vitro has been published although the effect was not long-lasting since removal of the compound was followed by a rapid increase in viral replication. The aim of this report is to describe the case of an HTLV-1 infected woman with HAM/TSP who was treated with GM1 for 6 months. The 40 year old individual was from Peru and started showing HAM/TSP symptoms (leg weakness, numbness, pain at the lower limbs and back) in 2010. In April 2013, she received Pregabalin (75 mg), although no improvement was observed. In December, the patient started to receive GM1, injected locally in her legs, according to the following schedule: 7 monthly applications combined with Acetilcarnitine and Citicoline (5 mg) followed by 3 months of resting. After 6 months, the treatment was suspended. Three months later, the patient kept on going with the Acetilcarnitine and Citicoline treatment. However, symptoms started to increase. The patient's description of the symptoms’ severity were consistent with proviral load (PVL) levels, quantified two months before the GM1 ganglioside treatment and 1, 5 and 8 months after. It could be suggested that porcine cerebral cortex GM1 monosialoganglioside applied monthly had positive short term effects reflected both in the amelioration of the symptoms and the PVL decrease. Further studies should be carried out in order to establish the role of ganglioside in HAM/TSP treatment.

